# Possible involvement of the autonomic nervous system in cervical muscles of patients with myalgic encephalomyelitis / chronic fatigue syndrome (ME/CFS)

**DOI:** 10.1186/s12891-021-04293-7

**Published:** 2021-05-05

**Authors:** Takayoshi Matsui, Kazuhiro Hara, Makoto Iwata, Shuntaro Hojo, Nobuyuki Shitara, Yuzo Endo, Hideoki Fukuoka, Masaki Matsui, Hiroshi Kawaguchi

**Affiliations:** 1 Orthopaedics and Spine Department, Tokyo Neurological Center, Toranomon 4-1-17, Minato-ku, Tokyo, 105-0001 Japan; 2 Matsui Hospital, Kan-nonji 739, Kagawa, Tokyo, 768-0013 Japan

**Keywords:** Myalgic encephalomyelitis / chronic fatigue syndrome (ME/CFS), Autonomic nervous system, Cervical muscle

## Abstract

**Background:**

Patients with myalgic encephalomyelitis / chronic fatigue syndrome (ME/CFS) sometimes present with stiffness of the cervical muscles. To investigate the pathophysiology of ME/CFS, this observational study compared patients with versus without recovery from ME/CFS through local modulation of the cervical muscles.

**Methods:**

Over a period of 11 years, a total of 1226 inpatients with ME/CFS who did not respond to outpatient care were enrolled in this study. All patients received daily cervical muscle physical therapy during hospitalization. Self-rated records documenting the presence or absence of ME/CFS, as well as the representative eight symptoms that frequently accompany it at admission and discharge, were compared. Pupil diameter was also measured to examine autonomic nervous system function involvement.

**Results:**

The recovery rate of ME/CFS after local therapy was 55.5%, and did not differ significantly by sex, age strata, and hospitalization period. The recovery rates of the eight symptoms were variable (36.6–86.9%); however, those of ME/CFS in the symptom subpopulations were similar (52.3–55.8%). The recovery rates of all symptoms showed strong associations with that of ME/CFS (*p* < 0.001). The pupil diameter was more constricted in the ME/CFS-recovered patients than in the ME/CFS-unrecovered patients in the total population and the subpopulations stratified by sex, age, and hospitalization period.

**Conclusions:**

There was a strong association between the recovery of ME/CFS and other related whole-body symptoms. The recovery of ME/CFS may be partly linked to amelioration of the autonomic nervous system in the cervical muscles.

**Trial registration:**

UMIN000036634. Registered 1 May 2019 - Retrospectively registered.

## Background

Myalgic encephalomyelitis / chronic fatigue syndrome (ME/CFS) is a serious, chronic, and complex disease that occasionally affects the lives of patients due to debilitating fatigue [1–4]. It is frequently accompanied by various symptoms, such as headache, cervical stiffness, vertigo, cardiovascular and gastrointestinal disorders, fever of unknown etiology, and psychological disorders. The high prevalence and low employment rates of patients with ME/CFS impose an enormous burden on society. The Institute of Medicine of the National Academy of Sciences reported that ME/CFS affects an estimated 2.5 million people in the United States and generates direct and indirect expenses of approximately $17–$24 billion annually [5].

Since ME/CFS is a heterogeneous condition with a complex and multifactorial etiology, reaching a conclusive diagnosis using the current methods is difficult. Although several studies have suggested the involvement of abnormal widespread metabolites [6–8], infection and neurological disorders [9], calcium ion channels [10], or anaerobic thresholds [11, 12]; the pathogenic mechanism of ME/CFS remains unclear. As such, patients are diagnosed through the exclusion of other conditions that could be responsible for the subjective symptoms with abnormalities across many domains [13, 14]. Thus, treatment remains symptom-based, multidimensional, and tailored to the needs of the individual patient [15, 16].

In our clinical experience, we have observed a potential trend of indefinite whole-body symptoms including ME/CFS occasionally coinciding with stiffness of the cervical muscles, and have therefore proposed a new medical concept called “cervical neuro-muscular syndrome” [17]. For the treatment of the indefinite symptoms, we tried local modulation of the cervical muscles. Among the physical therapies, low-frequency electrical stimulation [18, 19] and far-infrared irradiation [20] are reportedly effective at treating stiffness of the cervical muscles. A previous study of patients with whiplash-associated disorders showed that the combined application of these two physical therapies to the cervical muscles ameliorated not only local symptoms in the neck and shoulder, but also indefinite symptoms in the whole body [21]. Furthermore, a recent study of 1863 patients showed that therapies applied to the cervical muscles significantly improved the indefinite whole-body symptoms including headache, cervical pain or stiffness, vertigo or dizziness, palpitation, dazzling, nausea or stomachache, fever of unknown etiology, and depression [22].

We propose autonomic nervous system involvement as an underlying causative mechanism. This system, which regulates the unconscious actions of the body via the sympathetic and parasympathetic nerves, reportedly plays a role in myalgic disorders such as fibromyalgia and low back pain [23, 24]. In contrast to the sympathetic nervous system’s excitatory role under stressful situations, the parasympathetic nervous system oversees resting, recovery from stress, and maintenance of homeostasis. Several nuclei of the hypothalamus generate coordinated patterns of responses of the two systems to internal or social stressors [25]. The pupil light reflex is known to be a representative indicator which can be used to evaluate autonomic nervous system function. In this reflex action, the constrictor muscle of the pupil decreases the diameter of the pupil under control of the ciliary ganglion, which is activated and innervated by a preganglionic autonomic nerve fiber [26–28]. In a recent study, the preliminary pupil light reflex test performed in a subpopulation, suggested possible autonomic nervous system dysfunction in the cervical muscles of patients with whole-body symptoms [22].

To investigate the pathophysiology of ME/CFS, we performed the two cervical muscle physical therapies [18–20] in 1226 patients with ME/CFS and examined the relationships between ME/CFS recovery and the representative eight whole-body symptoms that frequently accompany it. Furthermore, to evaluate the possible involvement of the autonomic nervous system as an underlying causative mechanism, we also compared the changes of the pupil diameters between patients who showed ME/CFS recovery versus those who did not show ME/CFS recovery.

## Methods

### Study design

This study is an observational study which compared patients with versus without recovery from ME/CFS.

### Patients

Of the patients who visited our institutions between May 2006 and May 2017, and were diagnosed with ME/CFS according to Fukuda’s definition [13], we enrolled 1363 patients who could not be successfully treated as outpatients, and were therefore hospitalized. Outpatient care was variable and included pharmacological and behavioral strategies but did not include the application of physical therapies to the cervical muscles. Hospitalization was decided by consent between patients and physicians independently of the severity of ME/CFS. The main reasons for hospitalization were persistent symptoms that required more intensive treatments, as well as the need for detailed examinations of other organs. Recovery from ME/CFS was also defined according to Fukuda’s diagnostic criteria [13], mainly by amelioration of chronic fatigue and exhaustion. Discharge was decided by consent between patients and physicians independent of the ME/CFS recovery by the definition above [13], and was determined mainly by considerable improvement of symptoms of ME/CFS or related whole-body symptoms. Patients who were hospitalized for 5–120 days were enrolled.

### Intervention

All patients underwent low-frequency electrical stimulation and far-infrared irradiation applied to the cervical muscles for 15 min two or three times daily throughout the hospitalization period. No other treatments such as medication, injection, external fixation, or cervical traction were performed. A combination of silver spike point (SSP; Nihon Medix, Chiba, Japan) and pain topra (LCF-30; Celcom, Inc., Fukuoka, Japan) was used for the low-frequency electrical stimulation, while a CERAPIA 3300 (Nihon Medix, Chiba, Japan) was used for the far-infrared ray irradiation.

For all participants, the self-rated records on the medical interview sheets documenting the presence or absence of the representative eight symptoms that frequently accompany ME/CFS [1–4], including headache, cervical pain or stiffness, vertigo or dizziness, palpitation, dazzling, nausea or stomachache, fever of unknown etiology, and depression, were collected at admission and discharge. Pupil diameter was also measured at admission and discharge, using a binocular infrared pupilometer (Iriscoder Dual C10641; Hamamatsu Photonics, Shizuoka, Japan). Each patient provided informed consent.

### Statistical analysis

Statistical analyses were performed using SPSS 16.0 J for Windows. *P* values less than 0.05 were considered statistically significant; all reported *p* values were two-sided. As the sample size (*n* = 1226) was sufficient, the central limit theorem could be applied to confirm that the data were normally distributed and that violation of the normality assumption would not cause major problems [29]. Hence, the paired Student’s t-test was used to examine the difference in the number of symptoms at admission versus discharge. The difference in the number of patients with each symptom between admission and discharge was evaluated using the chi-square test. The unpaired t-test was used to compare means between the recovered and unrecovered ME/CFS groups. In the univariate and multivariate logistic regression analyses, all variables were force entered into the multivariate model. Forward stepwise multivariate logistic regression analyses were also performed. The best model was selected based on likelihood ratio tests.

## Results

### Flow and backgrounds of participants

**Fig. 1:**
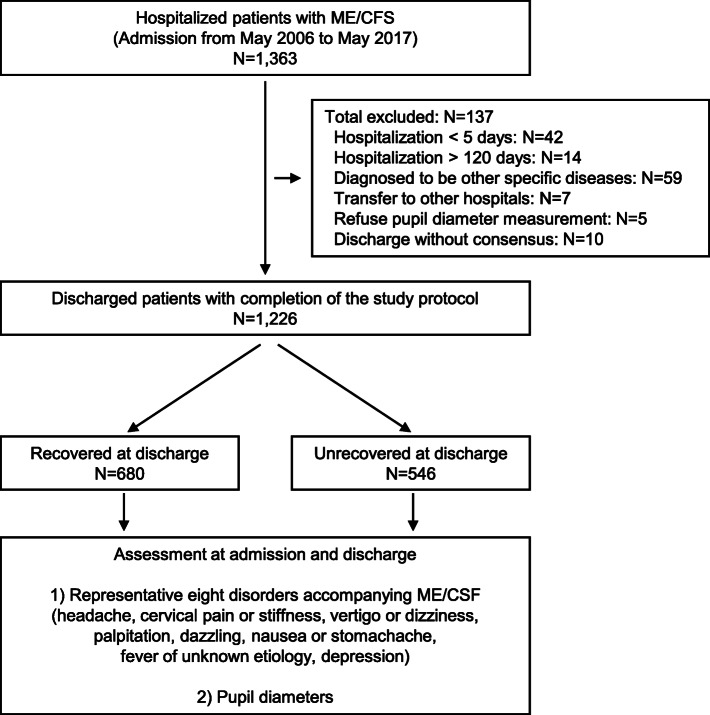
Flowchart of participant enrollment and study design

Figure 1 shows a flowchart of the patient enrollment process of the present study. A total of 1363 patients who were diagnosed with ME/CFS according to the definition above [13] and hospitalized in our institutions were initially enrolled in this study. Of this group, 137 were excluded after enrollment. This includes 42 who were discharged after less than 5 days; 14 who were hospitalized for more than 120 days; 59 who were diagnosed with specific diseases in other organs after admission (one of whom died during hospitalization); seven who were transferred to other hospitals for treatment of specific diseases; five who refused to undergo pupil diameter measurement; and 10 who discharged themselves from the hospital based on their own judgement with unknown reasons. After the removal of these patients from the study population, 1226 completed the study protocol. Of these patients, 680 (55.5%) were diagnosed as having recovered from ME/CFS at discharge, according to the definition above [13], while 546 (44.5%) remained unrecovered. The eight representative symptoms accompanying ME/CFS and the pupil diameters were assessed and compared between the ME/CFS-recovered and -unrecovered groups.

Table 1 shows the baseline characteristics of the 1226 participants (448 men, 778 women) with a mean age of 46.4 ± 16.3 years (mean ± standard deviation) and a mean hospitalization period of 62.5 ± 26.4 days. The recovery rate was not significantly altered by sex (men versus women), age strata (10–49 versus 50–89 years), or hospitalization period (5–60 versus 61–120 days) (*p* > 0.05).

**Table 1:** Baseline characteristics of study participants with versus without recovery

Variables	Number (%)	Number (%)	Number (%)	*P*-value
	Total	Recovered	Unrecovered	
	1226 (100.0)	680 (55.5)	546 (45.5)	
Sex				
Men	448 (36.5)	252 (56.3)	196 (43.7)	0.675
Women	778 (63.4)	428 (55.0)	350 (45.0)	
Age strata (years old)				
10–49	736 (60.0)	408 (55.3)	328 (44.7)	0.979
50–89	490 (40.0)	272 (55.5)	218 (44.5)	
Hospitalization period (days)				
5–60	518 (42.3)	276 (53.3)	242 (46.7)	0.188
61–120	708 (57.7)	404 (57.1)	304 (42.9)	

### Relationship between ME/CFS recovery and eight related symptoms

Among the representative eight symptoms accompanying ME/CFS [1–4], more than 70% of ME/CFS patients reported headache, cervical pain or stiffness, palpitation, dazzling, fever of unknown etiology, and depression; 43.1% reported vertigo or dizziness and 56.7% reported nausea or stomachache (Table 2, the leftmost column). The recovery rates of these symptoms in the total population, after physical therapies administered during hospitalization, were variable: more than 70% in patients with vertigo or dizziness, nausea or stomachache, and depression; 50–70% in patients with cervical pain or stiffness, palpitation, dazzling, and fever of unknown etiology; and 36.6% in patients with headache (Table 2, the leftmost column). The recovery rates of ME/CFS among patients with the eight symptoms were similar (52.3–55.8%) (Table 2, the second column from the left) to that of the total population (55.5%) (Table 1). Furthermore, the chi-square test (Table 2, the rightmost column) and logistic regression analyses (Table 3) between the recovery versus non-recovery of these symptoms clearly showed a strong association with ME/CFS recovery in all symptoms (*p* < 0.001). Among the symptoms, recovery from depression was most strongly associated with ME/CFS recovery (odds ratio, 13.70; Table 3).

**Table 2:** Number (percentage) of patients with versus without recovery according to the representative eight symptoms accompanying ME/CFS

	Total (*n* = 1226)	ME/CFS recovered	ME/CFS unrecovered	*P*-value
Headache	1162 (94.8)	648 (55.8)	514 (44.2)	< 0.001
Recovered	425 (36.6)	314 (73.9)	111 (26.1)	
Unrecovered	737 (63.4)	334 (45.3)	403 (54.7)	
Cervical pain or stiffness	1071 (87.4)	583 (54.4)	488 (45.6)	< 0.001
Recovered	606 (56.6)	414 (68.3)	192 (31.7)	
Unrecovered	465 (43.4)	169 (36.3)	296 (63.7)	
Vertigo or dizziness	528 (43.1)	285 (54.0)	243 (46.0)	< 0.001
Recovered	459 (86.9)	272 (59.3)	187 (40.7)	
Unrecovered	69 (13.1)	13 (18.8)	56 (81.2)	
Palpitation	960 (78.3)	502 (52.3)	458 (47.7)	< 0.001
Recovered	595 (62.0)	376 (63.2)	219 (36.8)	
Unrecovered	365 (38.0)	126 (34.5)	239 (65.5)	
Dazzling	979 (79.9)	536 (54.7)	443 (45.3)	< 0.001
Recovered	513 (52.4)	341 (66.5)	172 (33.5)	
Unrecovered	466 (47.6)	195 (41.8)	271 (58.2)	
Nausea or stomachache	695 (56.7)	378 (54.4)	317 (45.6)	< 0.001
Recovered	508 (73.1)	323 (63.6)	185 (36.4)	
Unrecovered	187 (26.9)	55 (29.4)	132 (70.6)	
Fever of unknown etiology	1045 (85.2)	554 (53.0)	491 (47.0)	< 0.001
Recovered	663 Z(63.4)	479 (72.2)	184 (27.8)	
Unrecovered	382 (36.6)	75 (19.6)	307 (80.4)	
Depression	894 (72.9)	470 (52.6)	424 (47.4)	< 0.001
Recovered	733 (82.0)	453 (61.8)	280 (38.2)	
Unrecovered	161 (18.0)	17 (10.6)	144 (89.4)

**Table 3:** Odds ratio (95% CI) of the recovery (vs. non-recovery) of each symptom to that of ME/CFS by logistic regression analysis

	n	Odds ratio	95% CI	*P*-value
Headache	1162	3.41	2.63–4.43	< 0.001
Cervical pain or stiffness	1071	3.78	2.92–4.87	< 0.001
Vertigo or dizziness	528	6.27	3.33–11.78	< 0.001
Palpitation	960	3.26	2.48–4.28	< 0.001
Dazzling	979	2.76	2.13–3.57	< 0.001
Nausea or stomachache	695	4.19	2.92–6.02	< 0.001
Fever of unknown etiology	1045	10.66	7.86–14.45	< 0.001
Depression	894	13.70	8.11–23.15	< 0.001

*CI* confidence of interval

### Pupil diameter test

We also examined the possible involvement of autonomic nervous system function by comparing pupil diameters of patients at admission and discharge (D-A) as well as the change ratio adjusted by the diameter at admission ([D-A]/A) (Table 4). In the total population, both change in pupil diameter (D-A = − 0.046 ± 0.633 mm; mean ± standard deviation) and change ratio ([D-A]/A = − 0.002 ± 0.123) decreased during hospitalization, suggesting that the physical therapies had contributed to improved autonomic nervous system function. These decreases were strongly evident in ME/CFS recovered patients (D-A = − 0.099 ± 0.700 mm, [D-A]/A = − 0.011 ± 0.134). However, there were no decreases, but rather increases, in the unrecovered patients (D-A = 0.020 ± 0.532 mm, [D-A]/A = 0.009 ± 0.107). A statistical analysis of the total population revealed a significant difference in the change in pupil diameter between the recovered and unrecovered groups (*p* = 0.001 for D-A, and *p* = 0.007 for [D-A]/A), suggesting an association between autonomic nervous system function and ME/CFS recovery.

**Table 4:** Pupil Diameters (mm) at Admission and Discharge

	Total				ME/CFS recovered				ME/CFS unrecovered				*P*-value D-A	*P*-value (D-A) / A
	Admission	Discharge	D-A	(D-A) / A	Admission	Discharge	D-A	(D-A) / A	Admission	Discharge	D-A	(D-A) / A		
Variables	5.440 (1.034)	5.394 (1.050)	-0.046 (0.633)	-0.002 (0.123)	5.480 (1.063)	5.380 (1.076)	-0.099 (0.700)	-0.011 (0.134)	5.391 (0.997)	5.411 (1.017)	0.020 (0.532)	0.009 (0.107)	0.001	0.007
Sex														
Men	5.621 (1.117)	5.541 (1.089)	−0.080 (0.621)	−0.008 (0.114)	5.672 (1.172)	5.520 (1.110)	−0.152 (0.666)	− 0.019 (0.119)	5.555 (1.042)	5.567 (1.064)	0.013 (0.548)	0.007 (0.104)	0.005	0.015
Women	5.336 (0.969)	5.310 (1.017)	−0.027 (0.640)	0.001 (0.129)	5.367 (0.977)	5.298 (1.047)	−0.068 (0.719)	−0.005 (0.142)	5.299 (0.960)	5.324 (0.980)	0.025 (0.523)	0.010 (0.109)	0.043	0.265
Age strata (years old)														
10–49	5.807 (0.914)	5.772 (0.901)	−0.035 (0.637)	0.001 (0.118)	5.853 (0.949)	5.764 (0.930)	−0.089 (0.687)	−0.008 (0.126)	5.750 (0.866)	5.782 (0.865)	0.032 (0.562)	0.011 (0.107)	0.010	0.029
50–89	4.889 (0.958)	4.827 (1.001)	−0.062 (0.629)	−0.006 (0.131)	4.920 (0.976)	4.806 (1.024)	−0.114 (0.721)	−0.015 (0.146)	4.850 (0.937)	4.853 (0.974)	0.003 (0.483)	0.005 (0.109)	0.040	0.101
Hospitalization period (days)														
5–60	5.505 (1.044)	5.455 (1.042)	−0.050 (0.606)	−0.003 (0.112)	5.594 (1.085)	5.459 (1.076)	−0.135 (0.674)	−0.018 (0.117)	5.404 (0.988)	5.450 (1.005)	0.047 (0.501)	0.014 (0.103)	0.001	0.001
61–120	5.393 (1.025)	5.349 (1.053)	−0.043 (0.653)	−0.001 (0.131)	5.401 (1.041)	5.326 (1.073)	−0.075 (0.717)	−0.005 (0.145)	5.381 (1.006)	5.380 (1.027)	−0.001 (0.554)	0.005 (0.111)	0.134	0.317

mean (standard deviation)

In subgroup analyses stratified by sex (men versus women), age strata (10–49 versus 50–89 years), and hospitalization period (5–60 versus 61–120 days), the decreases were reproducible in all subgroups except for the longer hospitalization (61–120 days) group in D-A (Table 4, the second column from the right), as well as the women, the younger generation (10–49 years) and the longer hospitalization (61–120 days) groups in (D-A)/A (Table 4, the rightmost column).

## Discussion

This study has shown that local therapy to the cervical muscles led to recovery in more than half of patients with ME/CFS. However, whether the cervical muscle is a possible target for treatment of ME/CFS remains unclear. In fact, the recovery rate of cervical pain or stiffness following the physical therapy was lower (56.6%) than that of other symptoms such as vertigo or dizziness, nausea or stomachache, and depression (> 70%) (Table 2, the leftmost column), suggesting that the mechanisms underlying the effect of the therapy might be other than direct modulation of the cervical muscles. The logistic regression analysis also showed that the odds ratio for the recovery of cervical pain or stiffness was lower than that of other symptoms, with that of depression being the highest (Table 3). This is consistent with the treatment response being due to psychological effects, rather than physiological effects. However, whether depression is a cause or consequence of ME/CFS remains unclarified, as previously reported [1, 4]. It is possible that the physical therapy initially improves psychological disorders such as depression through the cerebral limbic system, which then leads to recovery of the hypothalamus coordination of responses to the sympathetic and parasympathetic nervous systems [25]. Alternatively, the effect of the present therapy could possibly be indirect via concomitant central sensitization and/or myofascial trigger points of cervical soft tissues. It is also reported that ME/CFS symptoms could be related to hypermobility, intracranial hypertension, and craniocervical obstructions [30]. The present physical therapies, electrical stimulation and far-infrared irradiation, have also been reported to stimulate nerve regeneration and repair [31, 32], independently of direct muscle modulation. Further studies using objective and quantitative measurements of muscle stiffness, like the ultrasound elastography technique [33], may clarify whether the cervical muscle is a possible target for treatment of ME/CFS.

In this study, we measured pupil diameter without using a light stimulation as the indicator of autonomic nervous function. However, pupil light reflex under light stimulation is known to be more sensitive than measuring pupil diameter [26, 27], and has been used to test patients with clinical signs of autonomic nerve dysfunction such as those with Parkinson’s disease, Alzheimer’s disease, and diabetes mellitus [34–36]. Although this study initially aimed to measure pupil light reflex parameters under light stimulation, such as constriction rate and velocity, the institutional review board (IRB) did not allow us to deliver external stimulation that was not approved for the diagnosis or treatment of ME/CFS. However, in a recent study on patients with indefinite symptoms throughout the body, a subpopulation analysis of patients with dazzling exhibited a proportional improvement in the constriction rate and velocity of pupil diameter without stimulation by local therapies [22]. Hence, we assume that pupil diameter measured without light stimulation could represent the pupil light reflex parameters with stimulation as an indicator of autonomic nervous system function.

The canonical pathway that regulates pupil diameter is such that the ganglion cell axons project to the Edinger-Westphal nucleus in the midbrain, where the preganglionic parasympathetic neuron fiber in the oculomotor nerve is activated and commands the constrictor muscle of the pupil [28]. Although the oculomotor nerve does not pass through the cervical muscles, another non-canonical pathway via the afferent parasympathetic neuron fiber in the vagus nerve, arising from the brainstem and extending through cervical muscles down to the thoracic and abdominal viscera [37], may be involved in the regulation of pupil diameter. Also, the sympathetic nerve reportedly enters the orbit via the divisions of the trigeminal nerve and a plexus of nerves surrounding the ophthalmic artery, a part of which commands the constrictor muscle of the pupil as a long ciliary nerve [38, 39].

A recent report showed that chronic vestibular multicanalicular canalithiasis can be the trigger of symptoms in ME/CFS [40], suggesting the involvement of the vestibular nervous system. The cervicocollic and cervicoocular reflexes are afferent from the cervical muscles, and involve the vestibular nucleus complex which is the origin of the oculomotor nerve. Hence, the local therapy to the cervical muscles might possibly improve the oculomotor nerve function via the vestibular nervous system, and cause the amelioration of ME/CFS symptoms.

A limitation of the study is the use of Fukuda’s diagnostic criteria [13] which are soft outcome indicators with subjective reports by patients. Although the criteria were most popular for the definition of ME/CFS at the onset of this study (May 2006), at least in Japan, usage of the Canadian Consensus Criteria [41, 42], which is assumed to be reliable and objective consensus diagnostic criteria, would have been more suitable for the definition. The cardiopulmonary exercise test methodology for assessing fatigue and effort intolerance, which is a representative symptom of ME/CFS [11, 12], would also be an ideal tool for the definition. For the evaluation of the eight symptoms that frequently accompany ME/CFS as well, we used the self-rated records on the medical interview sheets documenting only presence or absence, which is subjective and qualitative. More quantitative variables with precise descriptions, such as a visual analogue scale, would have led to more accurate results.

The inpatient physical therapies performed in this study, two or three times daily for a mean of 62.5 days, are too costly for both health care providers and individuals. The development of more simple and feasible treatments is the next task. For the modulation of cervical muscles, we previously performed a prospective trial of the effects of an oral muscle-relaxant on ME/CFS. While the systemic modulation of muscle stiffness by the drug was somewhat effective at relieving local symptoms in the neck or shoulder, it had a minimal effect on whole-body disorders including ME/CFS (unpublished observation). Since we believe that local modulation of the cervical muscles independent of physical or medical intervention would effectively treat ME/CFS, we are now planning a prospective randomized controlled trial that will examine the effects of a topical muscle-relaxant poultice or ointment in patients with this disease.

## Conclusions

Although the effect of local modulation of the cervical muscles on ME/CFS remains unclear, there was a strong association between recoveries of ME/CFS and other related whole-body symptoms under the therapy. The recovery of ME/CFS may at least be partly through amelioration of the autonomic nervous system.

## Data Availability

The datasets used and/or analyzed during the current study are available from the corresponding author on reasonable request.

## Abbreviations

ME/CFS: Myalgic encephalomyelitis / chronic fatigue syndrome
FIR: Far-infrared irradiation
IRB: Institutional review board
SSP: Silver spike point
